# Don’t be *MIZ*guided, know where to grow!

**DOI:** 10.1038/s42003-023-05707-z

**Published:** 2024-01-09

**Authors:** Hannah Vahldick

**Affiliations:** 1 Department of Plant Biotechnology and Bioinformatics, Ghent University, Technologiepark 71, 9052 Ghent, Belgium; 2 VIB Center for Plant Systems Biology, Technologiepark 71, 9052 Ghent, Belgium

## Abstract

Plant organs shift their directional growth in response to environmental stimuli through tropisms. Arabidopsis roots exhibit positive hydrotropism (towards water) and negative phototropism (away from light). In a recent study, Pang and colleagues demonstrated that root phototropism is regulated by the activity of two proteins in the elongation zone that also play essential roles in hydrotropism.

**Figure:**
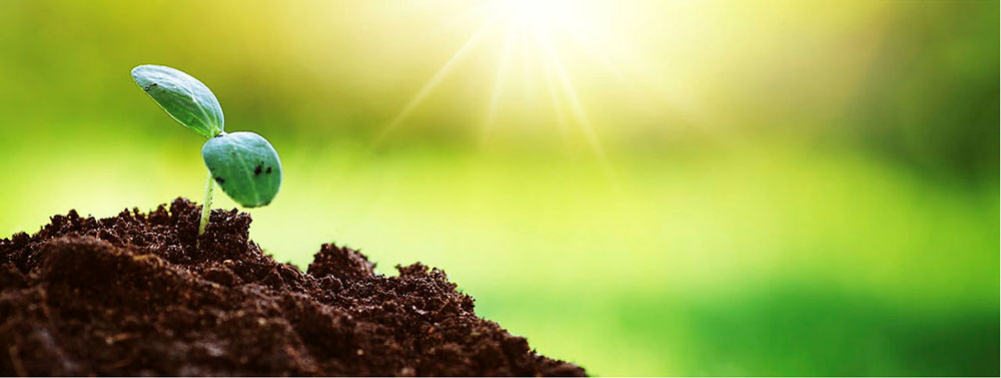
Candy1812, stock.adobe.com

Plants have evolved tropisms that allow for differential growth in response to environmental gradients, including light, gravity, water, touch, temperature, nutrients, and pathogens^1^, which enables them to grow towards resources (e.g., root positive hydrotropism) and avoid unnecessary stressors. Root hydrotropism is dependent on the genes *MIZU-KUSSEI1* (*MIZ1*) and *MIZ2*, whose names are derived from the Japanese words for “water” (mizu) and “tropism” (kussei)^2^. *MIZ1* encodes an endoplasmic reticulum membrane-associated protein with a largely unknown molecular function, while *MIZ2* encodes a guanine nucleotide exchange factor for ADP ribosylation factor (GNOM)^2–4^. In addition to lacking hydrotropic responses, roots of both *miz1* and *miz2* mutants also show substantially reduced negative phototropism—hinting at some underlying interaction between these tropic responses^2,3^.

A recent study by Pang et al. provides further evidence that root hydrotropism and phototropism are regulated to at least some degree by shared molecular pathways^5^. Building on previous findings that MIZ1 expression in the cortex of the transition and elongation zones is necessary for hydrotropism^6^, Pang et al. examined the hydro- and phototropic responses of tissue-specific complemented, transgenic *miz1* and *miz2* mutant roots. They showed that functional MIZ1-GFP expressed under a cortex-specific promoter is able to fully rescue the phototropic response of *miz1* plants, with roots demonstrating wild-type-like curvature in response to blue light exposure^5^. In contrast, expression of MIZ1-GFP in the root cap, meristem, epidermis, or endodermis is not sufficient to rescue phototropic responses in this mutant. In parallel, Pang and colleagues also demonstrated complete recovery of both hydrotropic and phototropic responses when *MIZ2/GNOM* is expressed in the epidermis, cortex, or stele of *miz2* roots, but not when it is expressed in the root cap or endodermis.

Pang and colleagues concluded that MIZ1 functions in the cortex of the elongation zone of roots, where it is necessary for a full phototropic response, while MIZ2/GNOM functions in the epidermis, cortex or stele to promote phototropism. These findings suggest that both phototropism and hydrotropism are at least partially mediated through homologous pathways. While it is logical that both MIZ1 and MIZ2/GNOM function in the same cell type (cortex), as it was previously demonstrated that MIZ1 activity depends on MIZ2/GNOM, MIZ2/GNOM’s tissue-specific ability to promote tropic responses in the epidermis or stele remains an intriguing mystery. Furthermore, additional work is needed to understand how MIZ1 and MIZ2/GNOM act on downstream factors to induce phototropism. For instance, future studies should focus on investigating if MIZ1 or MIZ2/GNOM act directly on key molecular components involved in phototropic signaling, such as the phototropin and phytochrome photoreceptors or the kinase PKS1.
